# The mycobacterial desaturase DesA2 is associated with mycolic acid biosynthesis

**DOI:** 10.1038/s41598-022-10589-y

**Published:** 2022-04-28

**Authors:** Rebeca Bailo, Anjana Radhakrishnan, Albel Singh, Makoto Nakaya, Nagatoshi Fujiwara, Apoorva Bhatt

**Affiliations:** 1 School of Biosciences and Institute of Microbiology and Infection, University of Birmingham, Edgbaston, Birmingham, B15 2TT UK; 2 School of Life Sciences, University of Warwick, Coventry, CV4 7AL UK; 3 Center for Research and Development of Bioresources, Organization for Research Promotion, Osaka Prefecture University, Sakai City, Osaka 599-8531 Japan; 4 Department of Food and Nutrition, Faculty of Contemporary Human Life Science, Tezukayama University, Nara City, Nara 631-8585 Japan

**Keywords:** Lipids, Biochemistry, Microbiology, Bacteria, Pathogens

## Abstract

Mycolic acids are critical for the survival and virulence of *Mycobacterium tuberculosis*, the causative agent of tuberculosis. Double bond formation in the merochain of mycolic acids remains poorly understood, though we have previously shown *desA1*, encoding an aerobic desaturase, is involved in mycolic acid desaturation. Here we show that a second desaturase encoded by *desA2* is also involved in mycolate biosynthesis. DesA2 is essential for growth of the fast-growing *Mycobacterium smegmatis* in laboratory media. Conditional depletion of DesA2 led to a decrease in mycolic acid biosynthesis and loss of mycobacterial viability. Additionally, DesA2-depleted cells also accumulated fatty acids of chain lengths C_19_-C_24_. The complete loss of mycolate biosynthesis following DesA2 depletion, and the absence of any monoenoic derivatives (found to accumulate on depletion of DesA1) suggests an early role for DesA2 in the mycolic acid biosynthesis machinery, highlighting its potential as a drug target.

## Introduction

**Figure 1:**
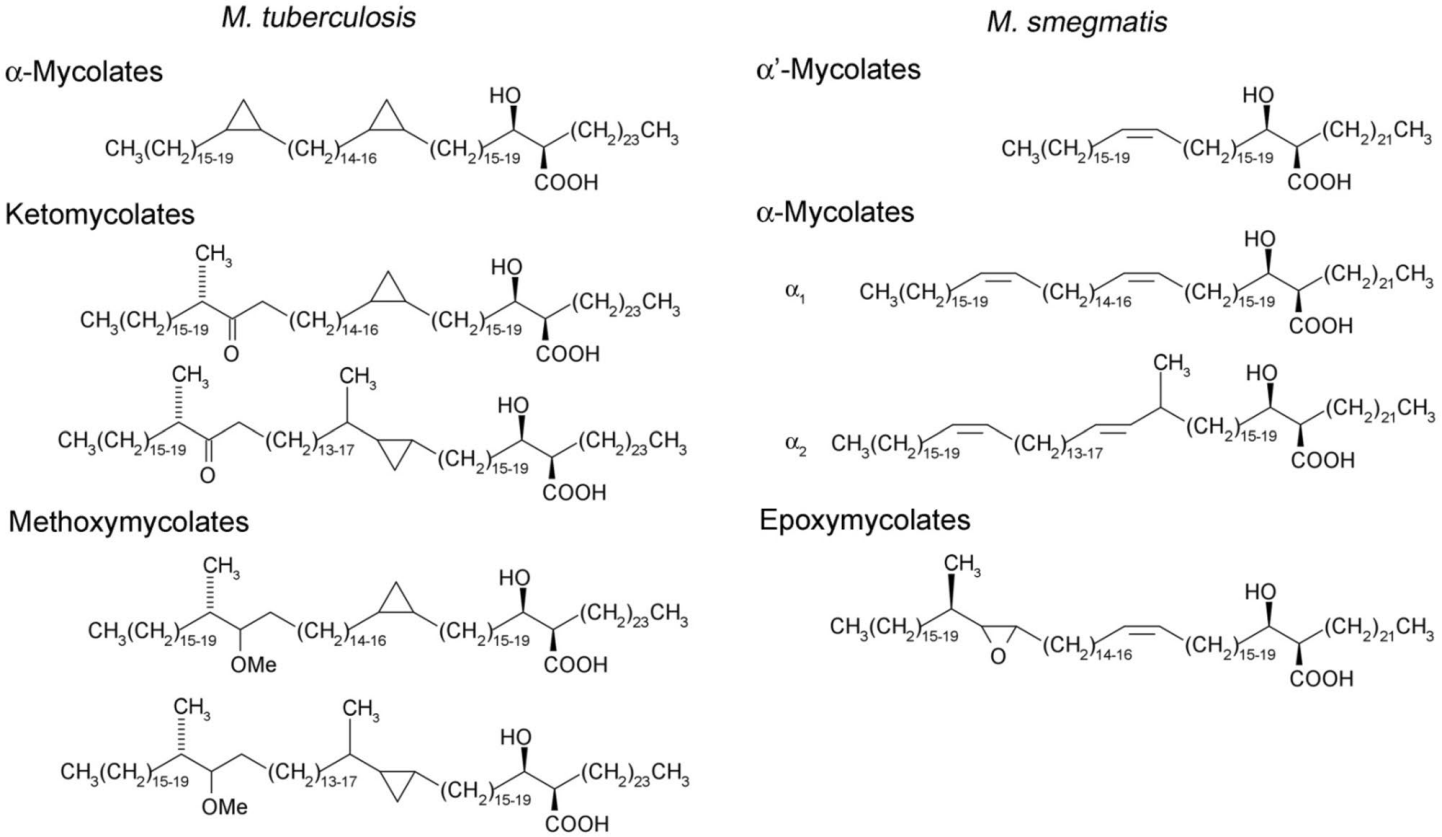
Structures of subclasses of mycolic acids found in *M. tuberculosis* and *M. smegmatis*.

Mycolic acids are long-chain fatty acids that are integral components of the cell walls of mycobacterial species and vital for survival and growth^1–3^. They are also produced by related genera such as *Corynebacterium*, *Nocardia* and *Rhodococcus*^4–6^. In pathogens such as the tuberculosis (TB)-causing *Mycobacterium tuberculosis*, mycolic acid biosynthesis is the target of the hallmark anti-TB drug isoniazid^7,8^. The mycolic acid core consists of a long merochain synthesised by a multienzyme Type II fatty acid synthase (FAS-II) by elongation of an ACP-bound acyl primer. A mycolic acid is made up of a merochain with an α-alkyl branch and a β-hydroxy group. The fast-growing saprophyte, *Mycobacterium smegmatis*, produces three subclasses, α-mycolates, shorter α′-mycolates and epoxy mycolates (Fig. 1). In *M. tuberculosis*, merochains contain modifications such as cyclopropanation, presence of keto groups or methoxy groups, generating chemical diversity resulting in three mycolate sub-classes (α, keto and methoxy, Fig. 1). These modifications are key drivers of pathology, particularly granuloma formation, and are vital for pathogenesis and persistence^9–11^. Two position-specific *cis*-double bonds on the merochain are substrates for subsequent modifying enzymes that introduce cyclopropane rings, keto groups or methoxy groups. The introduction of these double bonds is thought to occur during merochain elongation by the FAS-II enzyme complex^12^. Two aerobic desaturases, encoded by the *M. tuberculosis* genes *desA1* and *desA2*, were hypothesised to play a role in the desaturation of meromycolate chains. We have previously shown that *desA1* is an essential gene involved in desaturation of mycolic acids in *M. smegmatis*^13^. Conditional depletion of *M. smegmatis* DesA1 led to a relatively higher level of monoenoic derivatives of mycolates, and accumulation of truncated fatty acids (~ C_26_–C_48_), followed by a loss of mycolic acid biosynthesis, resulting in cell death. The accumulation of monoenoic derivatives suggested a potential role of DesA1 in adding the distal double bond in a growing merochain, though this could not be conclusively demonstrated in the absence detailed biochemical characterisation of the limiting amounts of the derivatives^13^. Interestingly, biophysical analysis of DesA1 revealed that it has a Ca^2+^ binding βγ-crystallin domain, suggesting a functional role for Ca^2+^ in DesA1 activity^14^. The role, if any, of DesA2 in mycolic acid biosynthesis is yet to be studied. However, there is some evidence that suggests that DesA2 is involved in mycolate desaturation. Solving of the DesA2 structure revealed that it is a homodimeric protein structurally related to plant acyl-ACP desaturases. Alterations in the metal-binding sites of DesA2 and two regions containing disordered residues in the interface between subunits suggested a structurally distinct substrate for this desaturase. Furthermore, we recently showed that MadR, a regulator of mycolic acid desaturation, acts as a repressor of not just *desA1*, but also *desA2*^15^. Here, we report genetic studies on *desA2* to probe its role in mycolic acid biosynthesis.

## Results

### Homologues of *desA2* are found in other mycolic acid-producing bacteria that utilise FAS-II

**Figure 2:**
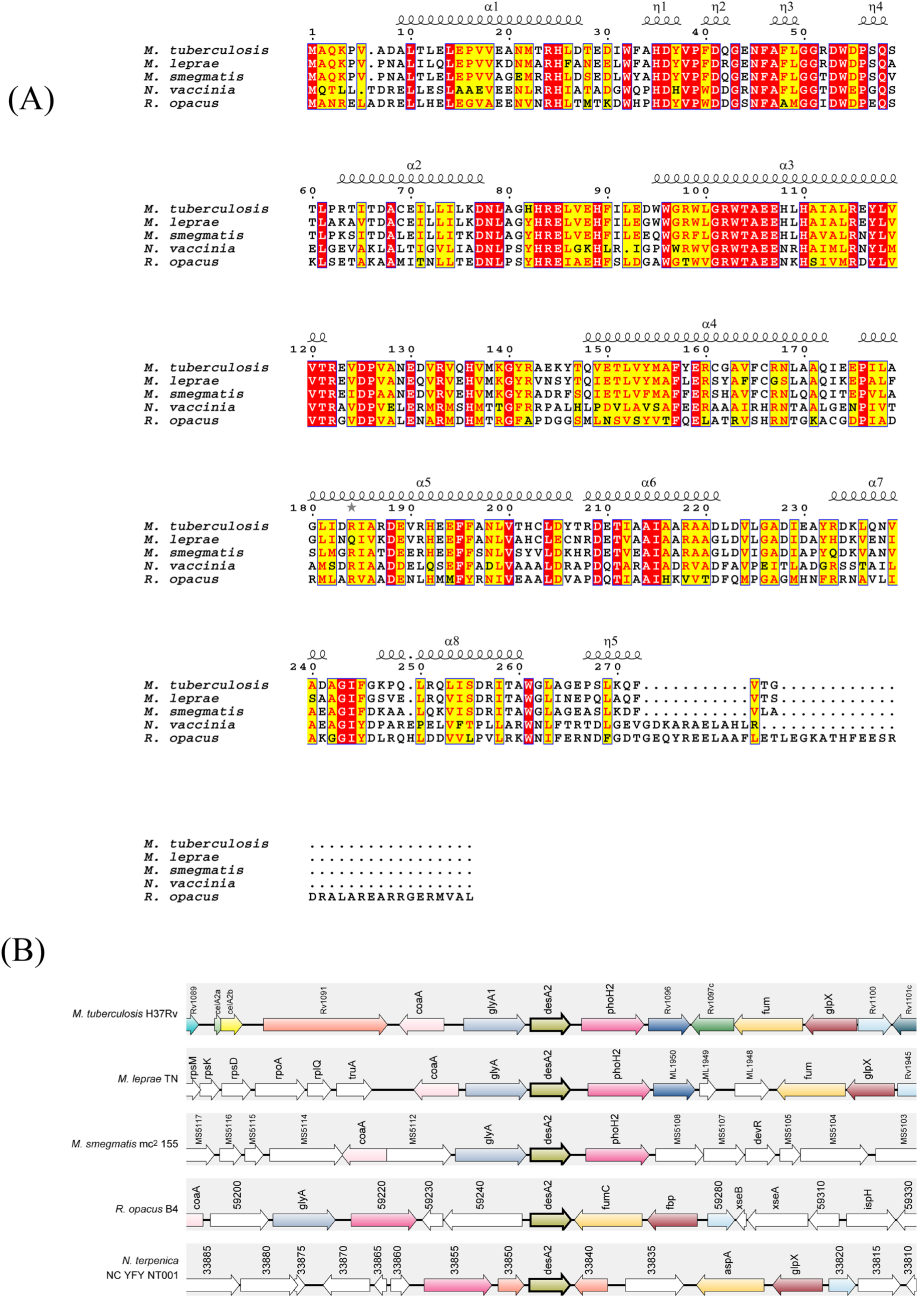
DesA2 is present in mycolic acid producing species that encode a FAS-II (**A**) Multiple sequence alignment of DesA2 orthologs (**B**) Genomic maps of regions containing *desA2*.

Homologues of *desA2* are present in other mycobacterial species including *M. smegmatis*, several non-tuberculous mycobacterial species (NTMs) and the obligate intracellular pathogen *Mycobacterium leprae* suggesting a core mycobacterial function for *desA2*. To further query a bespoke role for *desA2* in other mycolic acid-producing genera that synthesize long-chain mycolates using FAS-II, we used the *M. tuberculosis desA2* amino acid sequence as a query to search genomes of other mycolic acid-producing bacteria (the mycolata). Homologues of *desA2* were present in mycolata that produced long-chain mycolic acids, including species of *Nocardia* and *Rhodococcus* (Fig. 2). No homologues were found in the genomes of *Corynebacterium* species that produce shorter mycolic acid chains using FAS-I, suggesting *desA2* is a putative desaturase gene found only in FAS-II encoding mycolata.

### *DesA2* is an essential gene in mycobacteria

**Figure 3:**
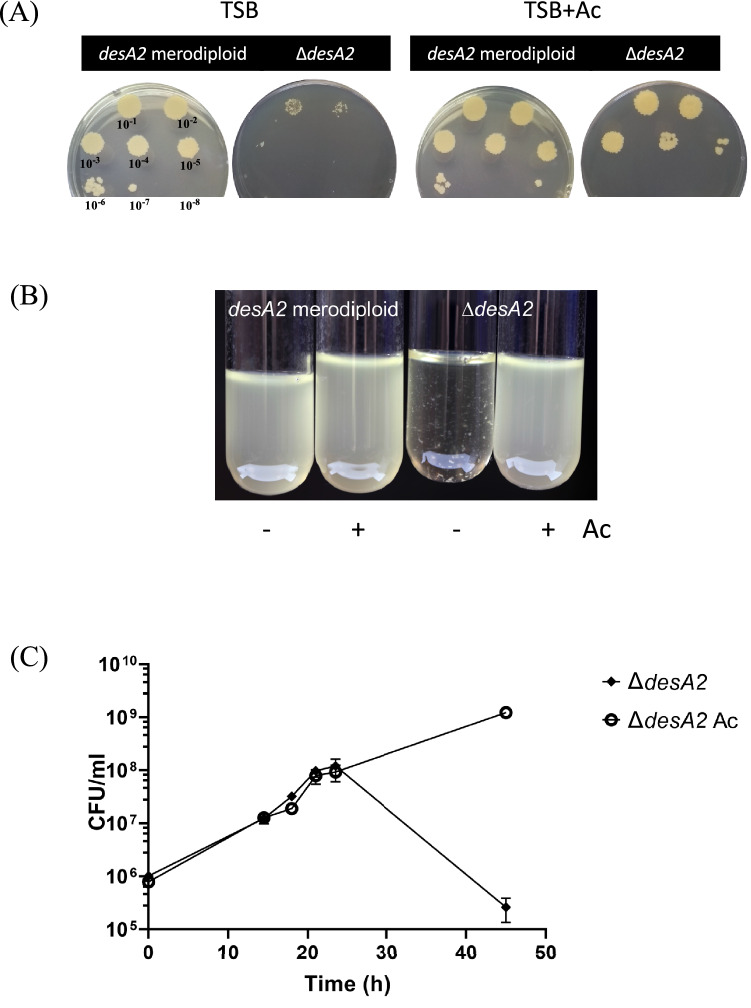
Growth of the ∆*desA2* mutant and the parental merodiploid strain on TSB agar plate (**A**) and 7H9 broth (**B**) in the presence or absence of acetamide. Serial dilutions spotted onto the TSB agar plates are indicated as a guide in the plate on the left (**C**) Growth curve of the ∆*desA2* mutant in 7H9 broth with or without acetamide.

We generated a recombinant phage designed to replace the native copy of *M. smegmatis desA2* with a hygromycin resistance cassette (*hyg*). Repeated attempts to generate a null *desA2* mutant in *M. smegmatis* by Specialized Transduction^16^ were unsuccessful, suggesting that *desA2* was essential for growth in laboratory media, an outcome often associated with mycolate biosynthesis genes. To demonstrate and validate the essentiality of *desA2* in *M. smegmati*s, we then transduced a merodiploid strain of *M. smegmatis* (containing an acetamidase promoter-driven, second integrated copy of *desA2*) with the knockout phage. Hygromycin resistant transductants were obtained only on plates containing the inducer, acetamide. One such transductant was confirmed by whole-genome sequencing to be a conditional *desA2* mutant, with the native copy of *desA2* replaced by *hyg*. These results demonstrated that *desA2* was an essential gene in *M. smegmatis*. A similar result was obtained with a *M. smegmatis* merodiploid containing acetamidase promoter-driven *M. tuberculosis desA2*, indicating that the *M. tuberculosis* homologue could compensate for the loss of function in *M. smegmatis*. The isolated conditional mutant termed ∆*desA2* was unable to grow on plates (Fig. 3A) or in broth lacking acetamide (Fig. 3B,C) confirming the essentiality of *desA2* in *M. smegmatis*. Saturation transposon mutagenesis screens in *M. tuberculosis* indicate that *M. tuberculosis desA2* is also an essential gene^17^.

### Loss of *desA2* impacts mycolic acid biosynthesis

**Figure 4:**
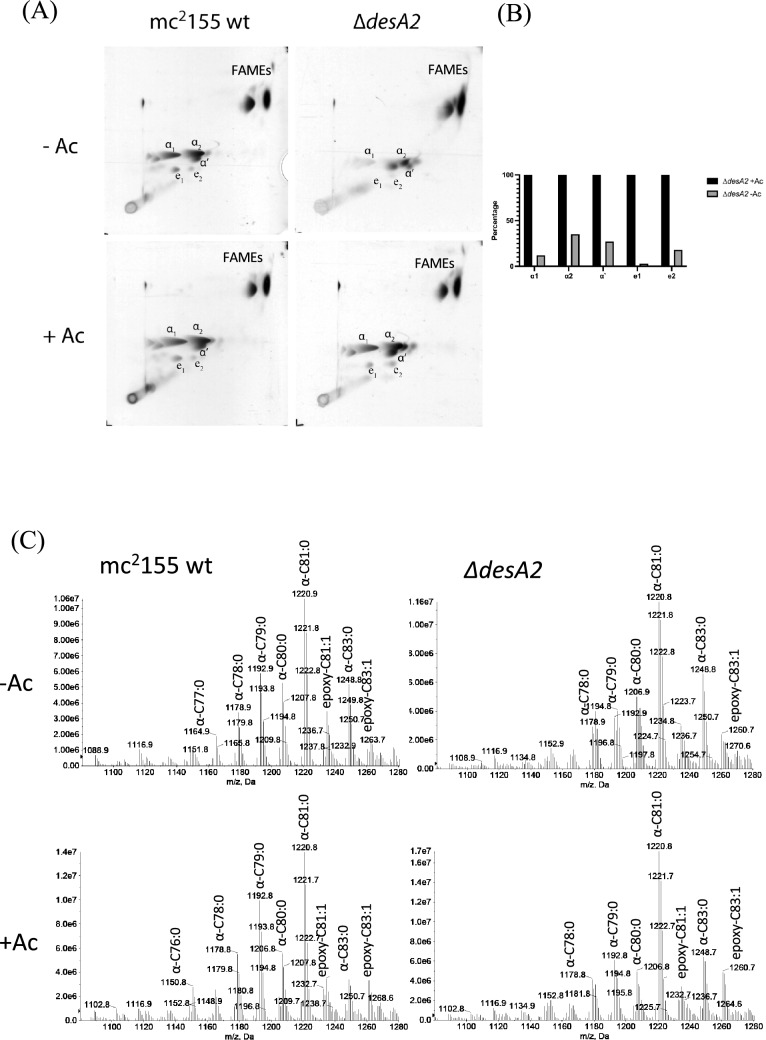
(**A**) Autoradiographs of two-dimension silver TLC plates used to separate [^14^C]-labelled fatty acid methyl esters (FAMEs) and mycolic acid methyl esters (MAMEs) extracted from the ∆*desA2* mutant. Ac; acetamide (**B**) Densitometric quantification of mycolic acid subspecies shown in (**A**). Comparative values shown for each subspecies, with those for − Ac cultures expressed as a percentage of those from + Ac cultures. (**C**) ESI–MS of MAMES extracted from the ∆*desA2* mutant.

**Figure 5:**
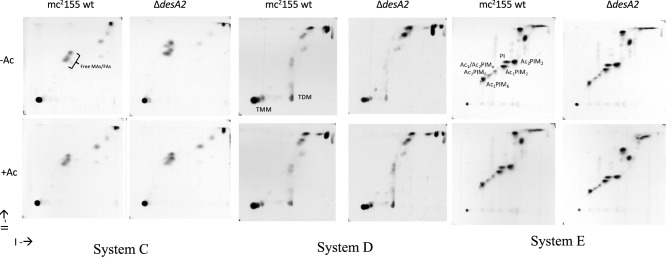
Autoradiographs of 2D-TLC analysis of apolar (Systems C, D) and polar lipids (System E) extracted from WT and ∆*desA2* mutant. Solvent systems C, D and E are as described by Dobson et al.^28^. *Ac* acetamide, *TMM* trehalose monomycolate, *TDM* trehalose dimycolate, *PIM* phosphatidyl inositol mannoside.

To further probe the potential role of *desA2* in mycolic acid biosynthesis, we analysed [^14^C]-labelled mycolic acid methyl esters (MAMES) extracted from cultures of the ∆*desA2* conditional mutant grown in the presence or absence of acetamide. Two dimensional (2D)-argentation TLC is a useful tool for analysing heterogeneity in desaturation (double bond) levels in mycolic acid sub-classes. The second TLC dimension separates saturated and unsaturated species in an AgNO_3_-treated section of the TLC plate, as the presence of double bonds retards migration in this dimension. Our previous studies using this method with the *desA1* mutant revealed the presence of faster migrating α-mycolic acid species on depletion of DesA1^13^. We now sought to assess the effects of depletion of DesA2 on mycolic acid biosynthesis and concurrently mycolic acid desaturation using this approach. Cultures of ∆*desA2* grown in the presence or absence of acetamide were pulsed with [^14^C]-acetic acid to label lipids. Mycolic acids were extracted from the cultures and converted to mycolic acid methyl esters (MAMEs) prior to separation by 2D-argentation TLC. ^14^C-labelled MAMEs were visualised by autoradiography. We were not able to observe any MAMEs that migrated faster in the second dimension, but depletion seemed to result in an overall reduction in mycolic acid biosynthesis (Fig. 4A,B, Uncropped images available as Supplementary Information). The effects were specific to mycolic acids and mycolic acid containing lipids (TMM and TDM) as the biosynthesis of other lipids was not affected during the early stages of DesA2 depletion (Fig. 5), ruling out any non-specific effects on mycolate biosynthesis. A parallel ESI–MS analysis of MAMEs extracted from unlabelled cells did not reveal any qualitative differences between mycolic acids from cultures grown in the presence or absence of acetamide (Fig. 4C) indicating the absence of any monoenoic intermediates like those observed for the conditional *desA1* mutant^13^. These data showed that the loss of DesA2 specifically led to a loss of mycolic acids, confirming a role for DesA2 in mycolic acid biosynthesis. Surprisingly, the levels of other fatty acyl methyl esters (FAMEs) appeared elevated suggesting their accumulation in the DesA2-depleted cells (Fig. 4A).

### Depletion of DesA2 leads to accumulation of fatty acids

**Figure 6:**
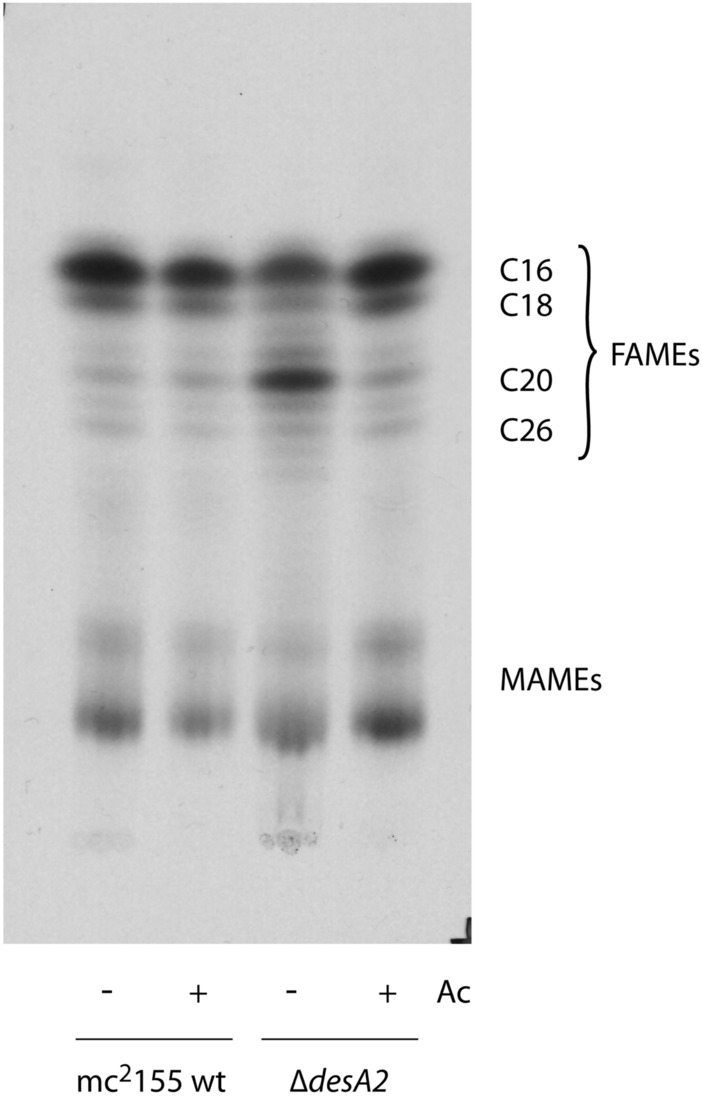
Autoradiograph of reverse phase TLC of [^14^C]-labelled fatty acid methyl esters (FAMEs) and mycolic acid methyl esters (MAMEs) extracted from the ∆*desA2* mutant. *Ac* acetamide.

**Figure 7:**
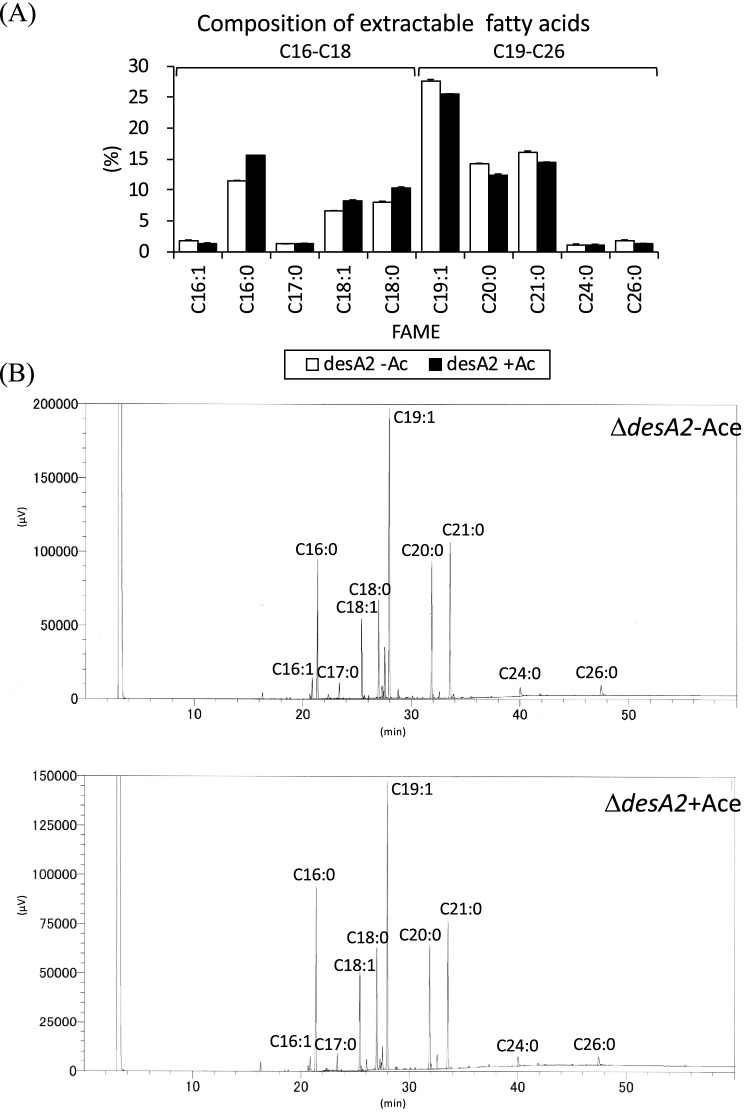
Molecular species compositions of extractable fatty acid methyl esters (FAMEs) from ∆*desA2* mutant as determined by GC. (**A**) GC analysis of the extracts from the ∆*desA2* mutant. (**B**) Relative quantities of FAME peaks comparing cultures grown in the presence or absence of acetamide.

We further probed the accumulation of FAMEs in the TLCs shown in Fig. 4A by analysing the FAMEs/MAMEs extract by reverse phase TLC, which enabled the separation of (relatively) short-chain fatty acids. Interestingly, conditional depletion of DesA2 led to the accumulation of fatty acyl methyl esters corresponding to C_19_–C_24_ (Fig. 6, uncropped image available as Supplementary Information). To determine if there were qualitative differences between the FAMEs extracted from the two culture conditions, we further probed the nature of these accumulating FAMEs in the DesA2-depleted cultures using GC. There were slightly higher levels of C_19_–C_24_ fatty acids in the Δ*desA2* conditional mutant grown in the absence of acetamide, compared to those seen in cultures grown in the presence of acetamide (Fig. 7). No unique species were identified in the acetamide-lacking cultures when compared to the GC spectra observed with extracts from the acetamide-containing cultures, suggesting that changes following depletion of DesA2 were a result of the accumulation of known mycobacterial fatty acids, rather than the accumulation of new classes of fatty acids.

## Discussion

*DesA1* and *desA2* encode the two putative aerobic desaturases in mycobacterial genomes. Similar to *desA1*^13^, we show here that *desA2* is also an essential gene in *M. smegmatis* and is associated with mycolic acid biosynthesis. However, the outcomes of desaturase depletion were very different for the conditional mutants of the two genes. While we detected a transient accumulation of minor mycolic acid-related species (likely monoenoic derivatives of α-mycolic acids) and free fatty acyl species of lengths ~ C_26_–C_48_ in the conditional *desA1* mutant, no such intermediates were observed on conditional depletion of DesA2. Instead, we saw the accumulation of C_19_–C_24_ fatty acids which are likely end products of the bimodal FAS-I^18^. The accumulation of these intermediate chain fatty acids mimics what is observed following an early and complete shutdown of mycolic acid biosynthesis ^19^ core components of FAS-II^12,20^. There may also be an alternative explanation for the accumulation of C_19_–C_24_ fatty acids in DesA2 depleted cells: they also represent the predicted mero chain lengths which would be synthesised prior to the addition of the first (proximal) double bond (Fig. 1). This, combined with the loss of mycolic acid biosynthesis and the absence of any monoenoic intermediates (unlike in the *desA1* mutant) following DesA2 depletion in the *M. smegmatis* conditional mutant suggests an early role for DesA2, likely the addition of the proximal (first) double bond on the growing merochain. Our studies with the conditional *desA2* mutant could not decipher further functional information about DesA2, but they prove its role in mycolic acid biosynthesis and highlight its potential as a new drug target within the mycolic acid biosynthesis pathway.

## Methods

### Chemicals

All solvents used were reagent grade from Sigma-Aldrich, Fisher Chemicals and Merk.

### Bacterial strains

*Mycobacterium smegmatis* mc^2^155 strains were grown in either tryptic soy broth (TSB, Difco) supplemented with 0.05% Tween-80 or 7H9 media (Middlebrook) supplemented with 0.05% Tween-80 and 0.2% glycerol. For the *M. smegmatis* Δ*desA2* conditional mutant, 0.2% acetamide was also supplemented (as described below). *Escherichia coli* TOP10 (Invitrogen) were cultured in LB (Difco). TSB-agar and LB-agar were prepared by adding 1.5% agar to TSB or LB prior to autoclaving. The antibiotic concentrations used for *M. smegmatis* and *E. coli* strains were 100 μg/ml for hygromycin (Invitrogen) and 50 μg/ml kanamycin (Sigma-Aldrich).

### Bioinformatics

Protein homology among the bacterial species was determined with Blastp. Then, sequence alignments were performed using the T-coffee^21^ web server under the mode PSI-Coffee and rendered with the EScript 3.0^22^ web server including the PDB: 1ZA0^23^ with the resolved structure of *M. tuberculosis* DesA2. *DesA2* genomic context in selected species was represented using SyntTax^24^ web server.

### Construction of *M. smegmatis* Δ*MSMEG5248* (Δ*desA2*) conditional mutant

The *M. smegmatis* mc^2^155::pMVacRv1094 ΔMSMEG5248 (Δ*desA2*) conditional mutant was generated using the genetic tool CESTET^25^. First, the *M. smegmatis* mc^2^155::pMVacRv1094 merodiploid *desA2* strain was constructed transforming the single-copy-integrating pMVacRv1094 vector into *M. smegmatis* mc^2^155 wt. pMVacRv1094 was generated by triple ligation of pMV306^26^ digested with XbaI and HindIII, the 2.7 kb fragment containing the acetamidase promoter from pSD26^27^ digested with XbaI and BamHI, and the PCR fragment including the *M. tuberculosis rv1094* gene obtained using the primers pMVacRv1094-Fw (TTTTTTTTAGATCTATGGCACAGAAACCTGTC) and pMVacRv1094-Rv (TTTTTTTTAAGCTTCTAGCCCGTGACGAATTG) and *M. tuberculosis* H37Rv genomic DNA as a template.

The mc^2^155::pMVacRv1094 merodiploid strain was then subjected to specialized transduction as previously described^16^ using the phage phΔMSMEG5248. Briefly, approximately 1 kb regions upstream and downstream of MSMEG5248 were amplified by PCR using the primers Ms5248LL (TTTTTTTTGCATAAATTGCCACGACCACGCACAAGTC), Ms5248LR (TTTTTTTTGCATTTCTTGCCCCACTGTTCTTCGAGGATG), Ms5248RL (TTTTTTTTCCATAGATTGGTCTTCTCCAACCTCGTCAGC) and Ms5248RR (TTTTTTTTCCATCTTTTGGGGATGTCCTTGCTCACCAAC) and cloned on either side of a hygromycin resistance cassette in the p0004S vector to generate the plasmid pΔMSMEG5248. The insertion of pΔMSMEG5248 into the temperature-sensitive mycobacteriophage phAE159^16^ produced the recombinant knockout phage phΔMSMEG5248. Transductants were selected on TSB-agar plates containing 150 μg/ml hygromycin and 0.02% acetamide at the non-permissive temperature of 37ºC. After confirmation of the gene replacement by PCR and whole-genome sequencing, one of the transductants was designated as Δ*desA2* and used for further analysis.

### Conditional depletion of *desA2* and determination of viable-cell counts

Δ*desA2* conditional mutant was grown in 3 ml TSB-T and acetamide up to an OD_600_ of 1. Then, bacterial cells were washed with PBS to remove traces of acetamide and resuspended in their original volume with TSB-T. 30 µl of the cell suspension was used to grow 2 cultures of 3 ml TSB-T with and without acetamide at 37 °C ON. 1:10,000 dilution of the same cell suspension was used to inoculate the Δ*desA2* conditional mutant in TSB-agar plates with and without acetamide, which were grown at 37 °C for 3 days. For CFU determination, Δ*desA2* was grown in TSB-T with and without acetamide at 37 °C and dilutions of the aliquots taken at 0, 14, 18, 21, 24 and 45 h were spread on TSB-agar containing acetamide. Plates were incubated at 37 °C for 48–72 h before counting CFU.

### Extraction of radiolabelled fatty acids and mycolic acids for Thin Layer Chromatography (TLC) analysis

WT and Δ*desA2* conditional mutant strains were grown in 50 ml of TSB-T, being the Δ*desA2* media supplemented with acetamide, up to an OD_600_ of 1. Cultures were washed with PBS and resuspended in their original volume with TSB-T. The WT was inoculated in 1:10 ratio in 2 flasks containing 20 ml of TSB-T with or without acetamide. In the case of Δ*desA2*, 20 ml of the resuspended culture were transferred to 2 fresh flasks, adding acetamide to one of them. Then, ^14^C-labelled acetic acid (PerkinElmer) was added to each culture at a concentration of 1 μCi/ml to be incubated at 37 °C ON. Bacterial cultures were harvested and washed with PBS to start the lipid extraction as described previously^28^. Briefly, apolar lipids from the pellets were extracted twice by adding 4 ml of petroleum ether (60–80 °C; Fisher Chemicals) to 2 ml of Methanol (Fisher Chemicals): 0.3% sodium chloride (Fisher Chemicals) (10:1), mixing on the rotator, centrifuging and transferring the upper layer containing apolar lipids to a new tube. For polar lipid extraction, 2.3 ml of chloroform (Fisher Chemicals): methanol: 0.3% sodium chloride (9:10:3) was added to the lower layer, mixed on the rotator and then centrifuged. The supernatant was transferred to a fresh tube and the pellets were extracted twice with 750 µl of chloroform:methanol: 0.3% sodium chloride (5:10:4). The pooled extracts were mixed with 1.3 ml of chloroform and 1.3 ml of 0.3% sodium chloride, centrifuged and the lower layer containing the polar lipids transferred to a new tube. Lipids were separated by 2D-TLC using solvent systems described by Dobson et al.^28^. The remaining delipidated cells and a polar lipids were exposed to alkaline hydrolysis^29^ with 5% tetrabutylammonium hydroxide (TBAH; Sigma-Aldrich) at 100ºC ON, followed by adding 4 ml dichloromethane (Fisher Chemicals), 50 μl iodomethane (Merck), 2 ml water, and mixing for 30 min. The upper phase was discarded after centrifugation, and the lower phase was washed thrice with water, evaporated and dissolved in diethyl ether (Fisher Chemicals). After centrifugation, the resulting Fatty Acid Methyl Esters (FAMEs) and Mycolic Acid Methyl Esters (MAMEs) in the supernatant were transferred to a new tube, evaporated to dryness and resuspended in chloroform: methanol (2:1). MAMEs aliquots (15,000 CPM) extracted from the delipidated cells and apolar lipids were analysed by two-dimensional silver ion argentation TLC^29^, where Silica Gel 60 F254 plates (Merck) were immersed in 10% aqueous silver nitrate (Acros Organics) and activated at 100 °C for 20 min. Once the aliquots were spotted, MAMEs were separated running twice in the first direction with hexane:ethyl acetate (19:1) and thrice in the second direction with petroleum ether (60–80 °C): diethyl ether (17:3). FAMEs aliquots (15,000 CPM) were developed once in chloroform: methanol (2:3) using reverse-phase TLCs. Autoradiograms were produced after exposing Carestream^®^ Kodak^®^ BioMax^®^ MR film for 3 days.

### Analysis of MAMES and FAMEs

Electrospray Ionization Mass Spectrometry (ESI/MS) was performed on a 4000 QTRAP LC–MS/MS System (SCIEX Corp. Tokyo Japan) with an Acquity UPLC H-class-Bio (Waters Corp., Tokyo Japan). In LC–MS experiments, an XTerra MS C18 Column (125 Å, 3.5 µm, 2.1 mm × 150 mm, Waters Corp.) was utilized for the separation of MAMEs. Methanol (Mobile phase A) and chloroform (Mobile phase B) were used for gradient elution. Initial conditions of the mobile phase at 90% A/10% B followed by a linear gradient to 10% A/90% B in 40 min. The Ion Spray voltage was maintained at 4.2 kV. The temperature was 600 °C for MAMEs. The column eluent was introduced into the Turbo Spray ion source of an ESI/MS system operated in the positive ion mode. The mass spectrum was acquired from m/z 700–1500 with a frequency of 1 scan/0.90 s for MAMEs. Typically, 10 μl of the sample was injected for analysis. Analyst 1.6.2 software (SCIEX Corp) was used for system control, data accumulation, and data analysis. FAME analyses were performed with a GC-2014 gas chromatograph (Shimadzu Corp. Ltd., Tokyo, Japan) equipped with a flame ionization detector and Equity-1 (30 m × 0.25 mm I.D., df 0.25 µm, capillary GC column; Supelco Inc., Bellefonte, Penn., USA) at a temperature ranging from 150 °C for 4 min to 250 °C at an increase of 4 °C/min, holding 3 min, and to 280 °C at an increase of 4 °C/min, holding 4 min. Each peak was identified by its retention time compared with those of a fatty acid methyl ester mixture (Supelco Inc.).

## Supplementary Information

Supplementary Information 1.

Supplementary Information 2.

Supplementary Information 3.
