# Supplementary figures and images for: The mycobacterial desaturase DesA2 is associated with mycolic acid biosynthesis
Source: Sci Rep. 2022 Apr 28;12:6943. doi: 10.1038/s41598-022-10589-y (PMC9050676; doi:10.1038/s41598-022-10589-y)

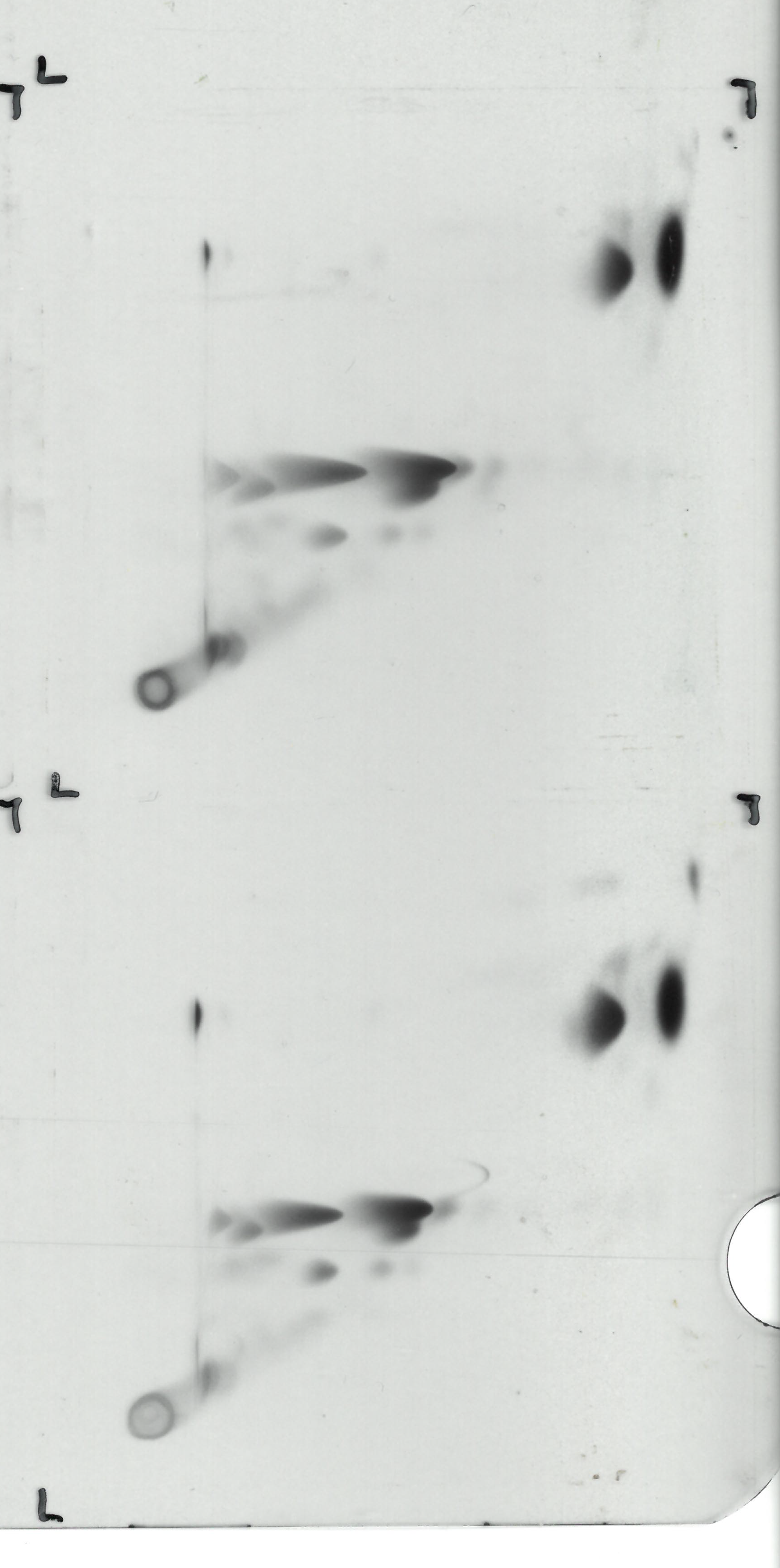

Supplement: Supplementary file 1 — Supplementary Information 1. [file 41598_2022_10589_MOESM1_ESM.tif]

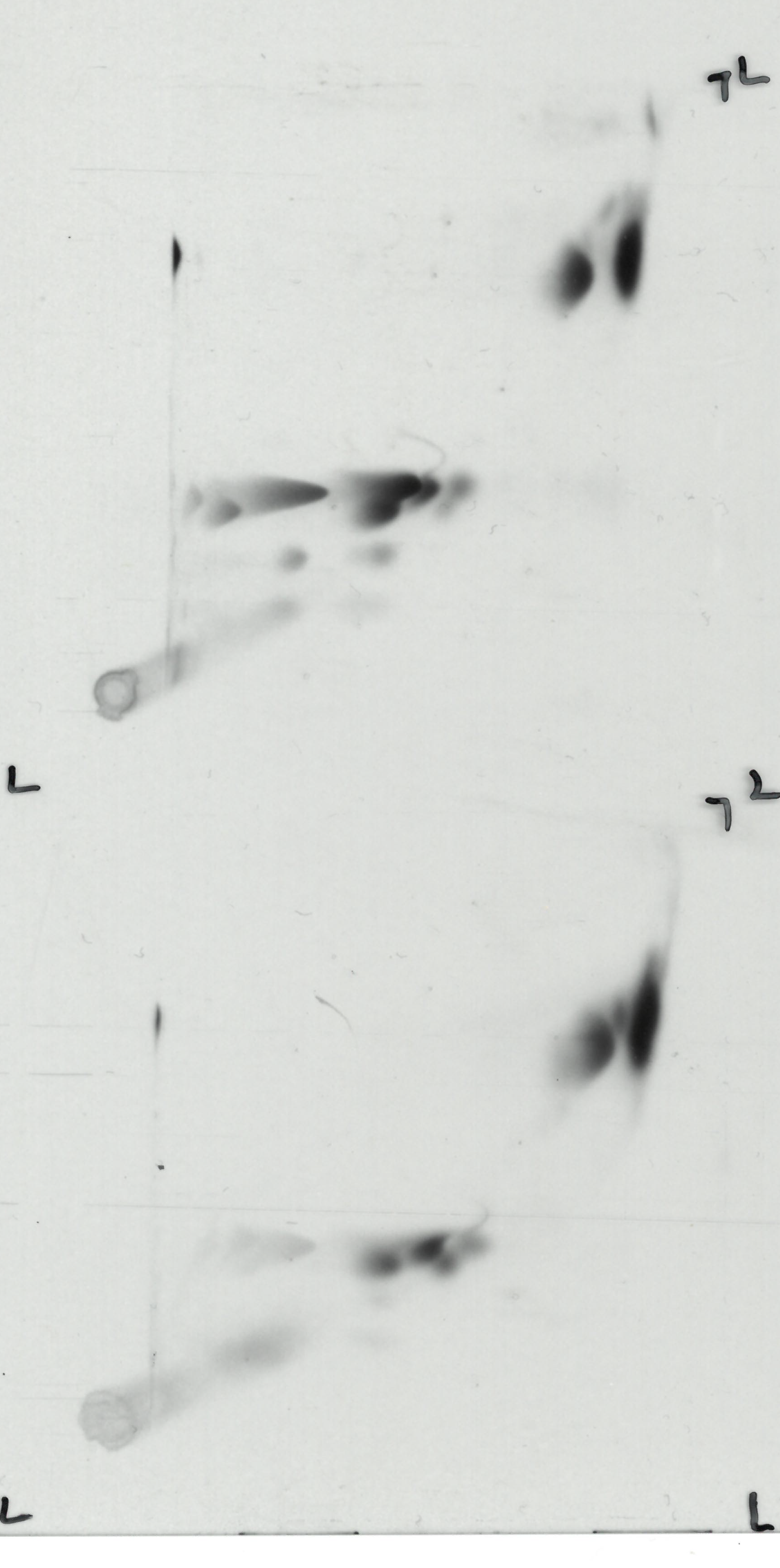

Supplement: Supplementary file 2 — Supplementary Information 2. [file 41598_2022_10589_MOESM2_ESM.tif]

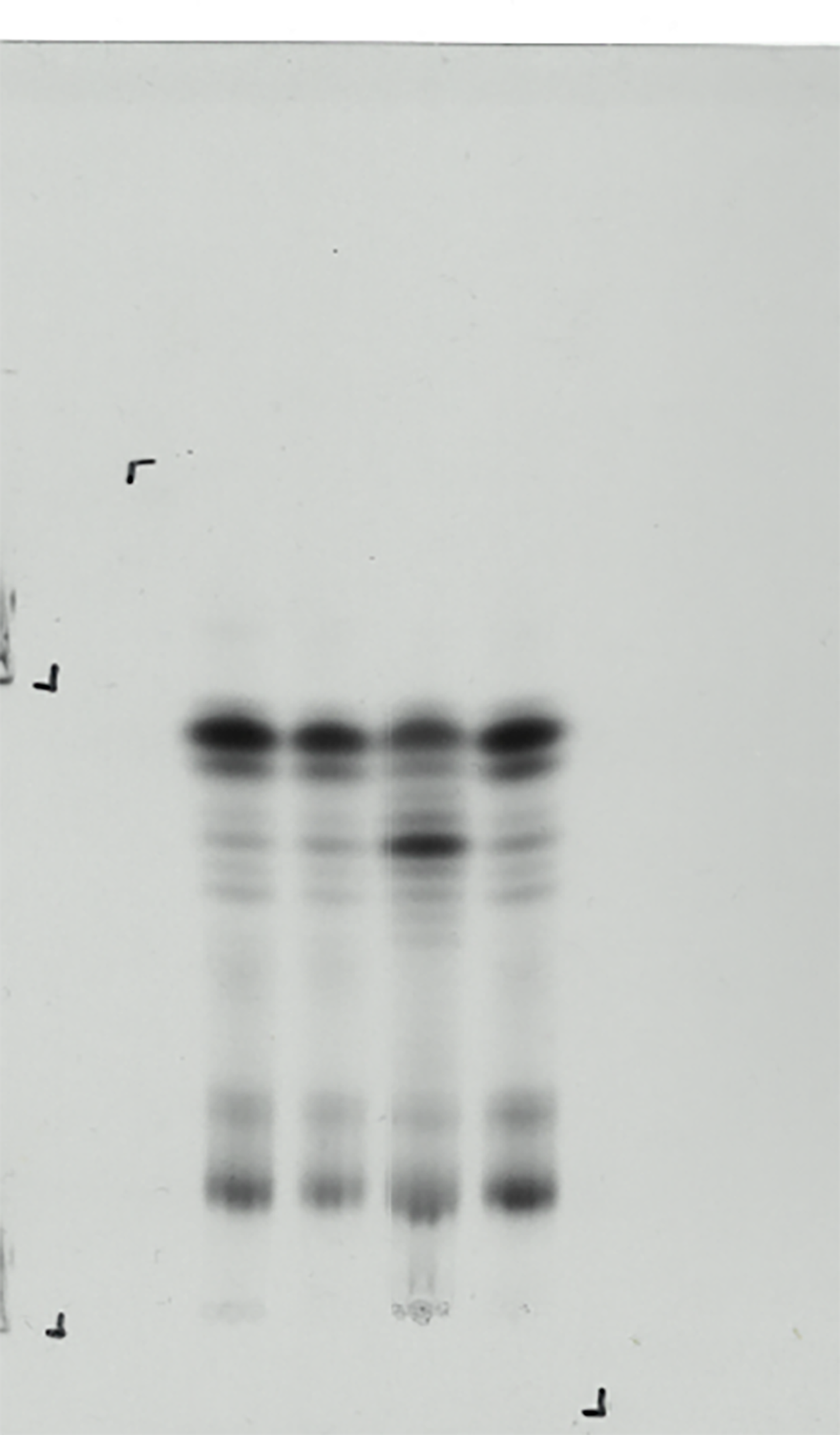

Supplement: Supplementary file 3 — Supplementary Information 3. [file 41598_2022_10589_MOESM3_ESM.png]
